# Therapeutic Potential of Mesenchymal Stem Cells and Their Products in Lung Diseases—Intravenous Administration versus Inhalation

**DOI:** 10.3390/pharmaceutics13020232

**Published:** 2021-02-07

**Authors:** Eleonore Fröhlich

**Affiliations:** 1 Center for Medical Research, Medical University of Graz, Stiftingtalstr 24, 8010 Graz, Austria; eleonore.froehlich@medunigraz.at; Tel.: +43-316-385-73011; 2 Research Center Pharmaceutical Engineering GmbH, Inffeldgasse 13, 8010 Graz, Austria

**Keywords:** mesenchymal stem cells, lung diseases, inhalation treatment, acute respiratory distress syndrome, extracellular vesicles, intravenous treatment, COVID-19

## Abstract

The number of publications studying the therapeutic use of stem cells has steadily increased since 2000. Compared to other applications, there has been little interest in the evaluation of mesenchymal stem cells (MSCs) and MSC-derived products (mostly extracellular vesicles) for the treatment of respiratory diseases. Due to the lack of efficient treatments for acute respiratory distress syndrome caused by infections with severe acute respiratory syndrome coronavirus 2 (SARS-CoV-2), the action of MSCs has also been studied. This review describes mode of action and use of MSCs and MSC-derived products in the treatment of lung diseases including the respective advantages and limitations of the products. Further, issues related to standardized production are addressed. Administration by inhalation of MSCs, compared to intravenous injection, could decrease cell damage by shear stress, eliminate the barrier to reach target cells in the alveoli, prevent thrombus formation in the pulmonary vasculature and retention in filter for extracorporeal membrane oxygenation. There is more feasible to deliver extracellular vesicles than MSCs with inhalers, offering the advantage of non-invasive and repeated administration by the patient. Major obstacles for comparison of results are heterogeneity of the products, differences in the treatment protocols and small study cohorts.

## 1. Introduction

Somatic cell therapy and tissue-engineered products belong to the advanced therapy medicinal products (ATMPs) [1]. Several products, Provenge^®^, MACI^®^, Chondrocelect^®^, Zalmoxis^®^, Alofisel^®^, KTE-X19^®^, Holoclar^®^ and Spherox^®^ have obtained marketing authorization but Provenge^®^, MACI^®^, Chondrocelect^®^ and Zalmoxis^®^ already withdrew their product from the market [2]. Alofisel^®^ contains allogenic adipose stem cells for local treatment of perianal fistula in M. Crohn. KTE-X19^®^ are autologous anti-CD19-transduced CD3+ cells for application in relapsed Mantle cell lymphoma. Holoclar^®^ are ex vivo expanded autologous human corneal epithelial cells including stem cells for treatment of limbal stem cell deficiency, a rare eye disorder. Spherox^®^ represents spheroids composed of autologous chondrocytes in a matrix to replenish chondrocyte defects in joints. Compared to the few diseases, in which stem cell products are used, the panel of diseases that could be treated with adult stem cells is broad and includes cancer, autoimmune diseases, cardiovascular diseases, ocular diseases, immunodeficiencies, neurodegenerative diseases, anemias, wound healing, metabolic diseases and liver diseases [3]. A list of the approved stem cell products is provided by Wilson et al. [4].

Mesenchymal stem cells (MSCs) are more often used in clinical trials than other adult stem cells. For pulmonary diseases, there were 82 trials with MSCs compared to 29 trials with other stem cells reported [5]. MSCs represent a heterogenous population of true stem cells and differentiation committed progenitor cells. They are or have been under evaluation in ~1000 clinical trials with main indication for neurological, joint and cardiovascular diseases [6]. Further, they find application in tissue reconstruction [7]. Conditioned media obtained from MSCs, which contains secretome, microvesicles or exosomes, is predominantly used for regenerative purposes, for example, stroke, brain/spinal cord injury, bone/cartilage defect, skin/hair regeneration, wounds, myocardial infarction, neurodegenerative diseases and liver failure [8]. Therapeutic applications of exosomes target mainly cancer, infectious diseases, cardiovascular diseases and neurodegenerative diseases [9].

This review will focus on the use of MSCs and MSC-derived products in lung diseases and will describe sources, mode of action and pharmaceutical aspects in the production of such products. Potential differences in the biological effects of these products upon intravenous and inhaled administration will be discussed.

## 2. Pulmonary Indications for the Use of MSCs and MSC-Derived Products

### 2.1. Description of Diseases

According to preclinical studies, MSCs may act beneficial in various respiratory diseases. Asthma is characterized by reversible airway obstruction, hyperresponsiveness of airways and airway inflammation [10]. Variable airflow limitation is caused by bronchial smooth muscle contraction, mucosal edema and formation of “mucus plugs.” The most common form is allergic asthma, where the release of histamine, leukotrienes and proteolytic enzyme cause airway obstruction. Histological hallmarks are inflammatory cells, particularly eosinophils, in the airways. Reduction of inflammation is a main therapeutic aim to slow down disease progression.

Chronic obstructive pulmonary disease (COPD) represents a heterogeneous disease, which is currently the third leading cause of death worldwide and expected to be the leading cause in 15 years [11]. Pathological findings include chronic inflammation, mucus hypersecretion, fibrosis, emphysema and airway obstruction [12]. Injury is mainly mediated by cytotoxic T cells and neutrophils.

Bronchopulmonary dysplasia (BPD) is a multifactorial disease of prematurity that causes impaired lung development. It occurs most often in low-weight infants born more than two months early. This disease, particularly in the first stages with formation of hyaline membranes, patches of atelectasis and lymphatic dilation, resembles acute respiratory distress syndrome (ARDS) and is characterized by the same set of biomarkers [13]. ARDS, also termed acute lung injury, is characterized by severe inflammation in the lungs and presents as severe hypoxemia and bilateral opacities on chest x-ray that are not explained by heart failure [14]. Clinical complications are caused by diffuse alveolar damage with transfer of protein-rich fluid and cells, mainly red blood cells, from the vessels to the alveoli, leading to interstitial edema and dysfunction of the air-blood barrier. ARDS may be caused by sepsis and trauma but bacterial pneumonia and viral infections specifically with coronavirus and avian influenza virus (H5N1) are associated with a high incidence of ARDS [15]. Tightness of the air-blood barrier is determined by intercellular junctions between alveolar cells and, to a minor extent, between endothelial cells. In ARDS the high permeation of neutrophils weakens the intercellular junctions [16]. Further decrease of the barrier is caused by mechanical ventilation with high tidal volumes and elevated airway pressure. While many of the indications for MSCs in the respiratory tract are chronic diseases, ARDS in its first phase is a life-threatening condition with an average mortality rate of 43% [17].

Both BPD and ARDS in the stage of tissue repair resemble lung fibrosis. Interstitial lung fibrosis (IPF) is the most common form of chronic progressive fibrosing interstitial pneumonia, occurring primarily in adults aged >60 years and limited to the lungs. Pro-inflammatory and non-inflammatory pathways lead to chronic epithelial injury and formation of fibrotic scars. Resident alveolar macrophages appear crucial in mediating the fibrosis.

Pulmonary arterial hypertension (PAH) is characterized by massive microvascular loss that causes increased blood pressure in the lungs and may develop in the course of diseases of the heart, lungs or pulmonary vessels. Vascular remodeling is associated to pulmonary airway obstruction and the main target of the therapeutic interventions.

For two additional lung diseases, gene editing or replacement methods by stem cell therapies to correct the gene defect in monogenic lung diseases may be successful. For these treatments, epithelial stem cells or induced pluripotent stem cells are more relevant than MSCs [18,19]. Hereditary pulmonary alveolar proteinosis (hPAP) is a rare monogenic respiratory disorder, characterized by abnormal accumulation of surfactant in alveolar macrophages and pulmonary alveoli. Surfactant clearance function by the alveolar macrophages is impaired by mutations in the genes encoding the granulocyte-macrophage colony-stimulating factor receptor (CSF2R) α or β chains [20].

Autosomal-recessive cystic fibrosis (CF) is due to mutation of the cystic fibrosis transmembrane conductance regulator (CFTR), a cellular membrane protein that acts on chloride ion channels in epithelial cells. The defect causes increase of mucus viscosity with mucus plugging, segmental atelectasis, bronchiectasis and recurrent lung infections [21]. Although CF is not a typical indication for MSCs, two clinical trials had been initiated. While one study has been withdrawn, the other study of safety and tolerability of MSCs in adults with CF has recently been completed.

### 2.2. Stem Cells in the Lungs

**Figure 1:**
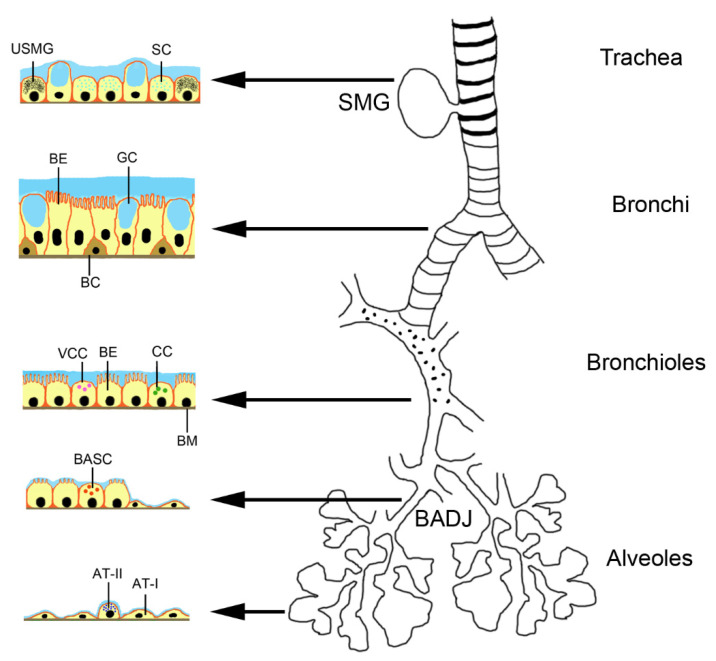
Location of stem cells and progenitor cells in the murine respiratory tract. Abbreviations: AT-I, alveolar epithelial cell type 1; AT-II, alveolar epithelial cell type 2; BADJ, broncholveolar duct junction; BASC, bronchoalveolar stem cells; BC, basal cell; BE, bronchial epithelial cell; BM, basal membrane; CC, Club cell; GC, goblet cell; SC, serous cell; SMG, seromucosal gland; USMG, undefined seromucosal gland duct cell; VCC, variant club cell.

Endogenous stem cells in the lungs can be activated by the disease and also may contribute to the paracrine effect of MSCs. Basal progenitor cells represent 30% of the pseudostratified mucociliary epithelium [22]. The TRP63+/KT5+ basal cells extend down to bronchioles of ~1 mm diameter in each lung with prominent inter- and intra-individual variations. They are classical stem cells in contrast to club cells, alveolar epithelial cells type II (AT II) and pre-alveolar type 1 cells, which arise from AT II cells upon lung injury. The system has been studied in much detail in mice and it is assumed that a similar situation is found in humans [23]. The seromucosal glands in the proximal region of the trachea contain serous cells, mucus cells and undefined seromucosal gland duct cells that can differentiate into serous cells, mucus tubule cells, collecting ducts cells, ciliated cells and myoepithelial cells (Figure 1). Basal cells in the pseudostratified bronchial epithelium of trachea and bronchi can replace ciliated cells and goblet cells. Variant club cells in the bronchiolar region can differentiate into club cells, ciliated cells and goblet cells. The basal stem cells of the broncholveolar duct junction can become club cells, ciliated cells, goblet cells and alveolar epithelial cells type (AT) I and II. AT II cells can differentiate into AT I and AT II cells.

## 3. Types of MSCs and MSC-Derived Products

### 3.1. Biological Characteristics of MSCs

The use of embryonic stem cells is not undisputed and regulation for their use vary between the countries [24]. Ethical concern of destroying embryonic cells and issues, such as tumorigenicity, risk of rejection and difficulty to obtain uniform differentiation in the target tissue, were the main reasons for preferring adult stem cells to embryonic stem cells. Adult stem cells, although they are less flexible regarding differentiation, available in smaller amounts and have a finite life span, are the preferred source for stem cell therapy. Due to their inherent plasticity, adult stem cells can differentiate into parenchymal cells. Sources for adult stem cells are blood, bone marrow, eye, brain, skeletal muscle, dental pulp, liver, gastrointestinal tract, pancreas and skin. MSCs have the advantage that, due to the lack of Major Histocompatibility Complex (MHC) class II and low class I expression, they are considered immune-evasive. They are not immune-privileged because allogenic MSCs caused a systemic inflammatory response 2h after infusion and induced antibody generation [25]. There may be even inter-species compatibility because human MSCs were effective in rats with PAH and in hyperoxic neonatal lung injury of mice [26,27]. MSCs can also activate the complement activation and induce thrombosis. However, since the reaction is usually mild and the retention of the MSCs in the body is low, allogenic MSCs can be used without major problems. Another issue of concern is the potential tumorigenic action of MSCs. The reason for the concern was sarcoma formation upon MSC administration reported in a mouse study [28]. Genetic instability (transformation) of the cells upon expansion in vitro was not experimentally verified and no tumor formation after repeated administration of MSCs to NOD mice and cynomolgus monkeys reported [29]. Although no tumor formation has been reported in humans, studies with long-term follow up are needed to enable a definite statement on their safety.

### 3.2. Modes of Action of MSCs

**Figure 2:**
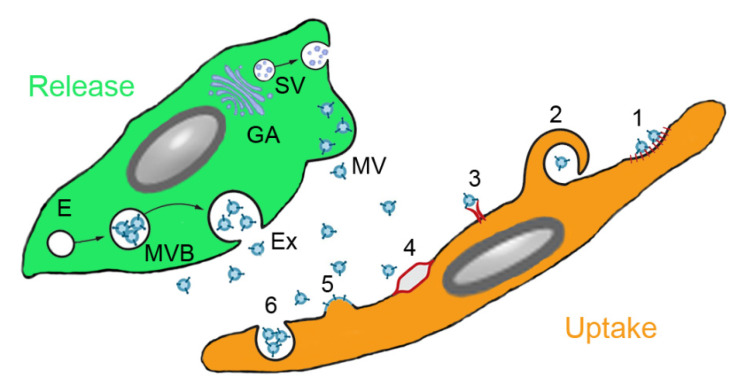
Paracrine secretion by mesenchymal stem cells (MSCs) and action on target cells. MSCs produce soluble molecules at the Golgi apparatus (GA) and release them as secretory vesicles (SV). Extracellular vesicles (EVs) are secreted either from endosomes (E) via multivesicular bodies (MVB) as exosomes (Ex) or by membrane budding as microvesicles (MV). EVs can be ingested by clathrin-or caveolin-mediated endocytosis (1), macropinocytosis (2), receptor-mediated uptake (3), lipid rafts (4), fusion with the plasma membrane (5) and phagocytosis (6) (routes according to [38]).

The mechanism of the beneficial effects of MSCs is not completely understood but several modes of action have been identified. Firstly, MSCs may differentiate to replace the damaged cells. This mechanism has been reported for renal tubular epithelial cells, epidermal keratinocytes and endothelial cells [30]. Integration into the walls of endothelial vessels has been observed after intraarterial injection of MSCs in the rat cremaster muscle microcirculation model [31] and in a similar way, MSCs delivered directly into the lung may also integrate into the alveolar epithelium [32]. Alternatively, cell fusion may take place because the very low frequency of cell fusion is increased in pathologic conditions. Alterations of the lipid bilayer upon inflammation are hypothesized as potential mechanism. Polymer chain reaction analysis from organs of patients, who received MSCs for various indications, showed that cell fusion contributed to the beneficial effect of MSCs, although to very low extent [33]. MSC donor DNA was detected in one or more tissues at levels of 1‰ to 1% of the cells. A study on co-culture of heat-shocked small airway epithelial cells and MSCs reported that up to 1% of the MSCs fused with epithelial cells [34]. Organelle transfer (mitochondria, lysosomes) may occur via thick (0.7 µm) tunneling nanotubes [35] from MSCs to macrophages, bronchial epithelial cells and alveolar epithelial cells. However, based on the fact that survival of MSCs in the body is short and that alginate-encapsulated MSCs acted similar to not encapsulated MSCs, it is assumed that MSCs act mainly by paracrine action [36]. This action may occur either by soluble molecules or by proteins and microRNAs (miRs) contained in extracellular vesicles (EVs). It is not easy to differentiate between soluble and vesicle-enclosed molecules because the isolation method of the EVs does not allow a good separation between the two. EVs can be released upon stimulation or after cell disruption due to shear stress in the blood circulation [37]. Paracrine interaction of MSCs with target cells is illustrated in Figure 2. Soluble factors are transported from the Golgi stacks as secretory vesicles to the plasma membrane, where they are released. Microvesicles are released by membrane budding and exosomes originate from endosomes and multivesicular bodies. Uptake by the target cells occurs either by clathrin- or caveolin-mediated endocytosis, macropinocytosis, receptor-mediated uptake, lipid rafts, fusion with the plasma membrane and (for phagocytic cells) phagocytosis.

**Figure 3:**
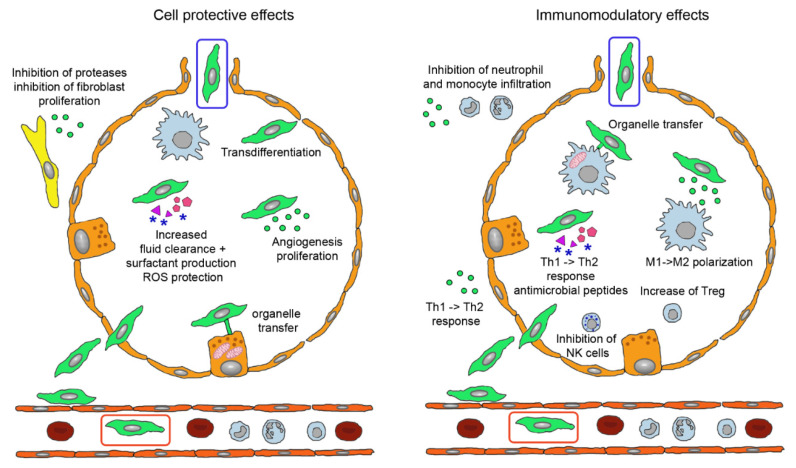
Biological effects of mesenchymal stem cells (MSCs) administered by intravenous (green cell in red box) and inhalation route (green cell in blue box). Injected MSCs have to extravasate and either locate in perivascular position or permeate the epithelial layer to reach the alveolar lumen. They perform tissue-protective and anti-fibrotic effects (**left**) by integration into the epithelial layer, release of extracellular vesicles (EVs) containing Ang-1, KGF, HGF, VEGF, EGF, mRNAs, miRs, membrane components and DNA for anti-apoptotic effects, increase of lung fluid clearance, epithelial cell proliferation and angiogenesis, transfer of mitochondria to alveolar epithelial cells via nanotubules and secretion of cytokines and other proteins. EVs containing inhibitors for metalloproteinases and for fibroblast proliferation act in the perialveolar space. Immunomodulatory functions (**right**) comprise organelle transfer to alveolar macrophages via nanotubules, release of TGF-β, PGE2, IDO, IL-10 to decrease T cell activation, TGF-β and HLA-G5 to stimulate Treg, PGE2 and TSG-6 to inhibit M1 activation and secretion of antimicrobial proteins LL-37 and lipocalin-1. The involved mediators either are contained in EVs or present as soluble factors. Abbreviations: Ang-1, angiopoietin-1; EGF, epithelial growth factor; HGF, hepatocyte growth factor; HLA-G5, human leukocyte antigen G5; IDO, indoleamine 2,3-dioxygenase; IL-10, interleukin 10; KGF, keratinocyte growth factor; LL-37, cathelicidin; miR, micro RNA; PGE2, prostaglandin E2; Treg, regulatory T cells; TGF-β, tumor growth factor-beta; TSG-6, TNF-stimulating gene 6; VEGF, vascular endothelial growth factor.

MSCs acted beneficial in asthma by release of EVs containing miRs to inhibit Th2 cells, in IPF by combination of inhibition of Th2 cells, stimulation of regulatory T cells (Treg) and inhibition of leucocyte infiltration via secretion of interleukin (IL)-1RA. Therapeutic effect of MSCs in COPD include secretion of epithelial growth factor (EGF), hepatocyte growth factor (HGF), keratinocyte growth factor (KGF), vascular endothelial growth factor (VEGF) to inhibit alveolar epithelial cell damage and apoptosis and release of EVs containing miR-100, miR-146a and miR-146-5b [39]. Effects of MSCs have been studied particularly in ARDS and can be grouped into improvement of the epithelial barrier by protection of epithelial cells, tightening of the intercellular functions, improvement of clearance function and decrease of inflammation by immunomodulatory effects (Figure 3) [40].

The contributions of the specific molecules and mediation by free or membrane-enclosed molecules differ between the studies. The majority of the reported effects, namely restoration of epithelial and endothelial function by increased cellular repair and decreased rate of apoptosis, higher surfactant production, increased resorption of lung fluid, restoration of tight junctions and reduced fibrin production may be mediated by EVs. EVs are also supposed to be involved in reduction of pro-inflammatory and enhancement of anti-inflammatory cytokine secretion, reduction of neutrophil infiltration and M2 polarization of alveolar macrophages [41]. Preclinical data identified cell-to-cell contact via programmed death-1 for MSC-T cell interaction, changes in amino acid and lipid metabolism by indoleamine 2,3-dioxygenase (IDO) expression, prostaglandin E2 (PGE2) production, tumor growth factor-beta (TGF-β) and HGF and increased expression of leukocyte protease inhibitor via EGF and HGF as important mechanisms [42]. Epithelial repair was mediated mainly by angiopoietin-1 (Ang-1), TNF-stimulating gene 6 (TSG-6) and lipoxin A4 (LXA4) and enhanced alveolar fluid clearance through activation of epithelial sodium channel by KGF [43]. Antimicrobiotic effects of MSCs in bacterial ARDS included increased phagocytosis of macrophages through mitochondria transfer, increased release of cathelicidin (LL-37) and of IL-10 and decreased tumor necrosis factor alpha (TNF-α) secretion mediated by PGE2 and LX4.

In bacterial/lipopolysaccharide (LPS)-induced ARDS, EVs containing Ang-1 and miR30b-3p for inhibition of epithelial damage are released by MSCs. miR-145, mitochondria and KGF stimulate oxygen consumption and phagocytosis of macrophages. MSCs inhibit secretion of IL-6 and TNF-α secretion, stimulate TSG-6 and LL-37 secretion and inhibit leukocyte invasion [39]. MSCs display on the one hand anti-inflammatory properties but, on the other, cause positive effects in animal models for bacterial pneumonitis. Preservation of neutrophilic granulocytes activity as the first line defense is likely and transfer of mitochondria to macrophages may make them more energetic and active in antibacterial defense. Rather than a standard release profile of MSCs the described mechanisms represent a panel of potential mechanisms, which can vary according to type and generation of MSC and to disease condition.

### 3.3. MSC-Derived Products

Conditioned media or secretome is a mixture of all organic and inorganic products secreted by cells. Its composition is similar to plasma, which contains free and vesicle-bound molecules mainly released from epithelial cells, endothelial cells and blood cells. Isolation of EVs from MSC-derived secretomes have been used in preclinical studies of lung diseases, where conditioned media from bone marrow-derived MSCs of rat, murine and human origin acted beneficial in murine and rat models of ARDS, murine asthma, murine and rat BPD/hyperoxia and rat fibrosis models [44]. Efficacy was obtained upon administration by intratracheal, intravenous, intranasal and intraperitoneal route. The priming of MSCs caused variable effects; while priming of MSCs by culture in hypoxia did not increase the efficacy of the secretome in ARDS model, addition of a Toll-like receptor 3 agonist had a positive effect. Differences between soluble proteins, different vesicles and whole cells may be related to disease-specific requirements: delivery of VEGF is most relevant in BPD, while fibroblast growth factor 2 (FGF-2) is the most important molecule in the treatment of COPD [45].

There are presently no approved MSC-conditional media but convalescent plasma has been used in the treatment of several virus-based diseases, namely for the Spanish Flu pandemic and infections with the SARS, MERS and influenza virus [46]. Plasma in addition to serum proteins (albumin and immunoglobulins) contains between 10^7^ and 10^9^ EVs/mL plasma in healthy individuals [47]. The reported beneficial effects in these diseases stimulated clinical trials in severely ill COVID-19 patients. Results from the several small case studies and the large trails, however, did not show mortality benefit or reduced progression to severe disease in patients admitted to hospital with moderate COVID-19 [48]. There is the possibility that concentrations both of transfused antibodies and of EVs was too low to induce the desired effect.

EVs are hypothesized to represent the most important active component of the secretome, although it is difficult to differentiate between soluble and membrane-enclosed molecules. EVs is a broad term to describe different types of vesicles secreted from cells. The function is communication between cells but they may also act as dustcarts. Exosomes are vesicles released by exocytosis of multivesicular bodies whereas ectosomes are assembled vesicles released at the plasma membrane [49]. Other classification is based on size with apoptotic bodies (0.1–5 µm), microvesicles (100–1000 nm) and exosomes (35–120 nm) as the main groups. Exosomes are further classified into small (60–80 nm), large (80–120 nm) and nanosized (~35 nm) vesicles [50]. The three types of EVs differ not only regarding size but also by origin, surface markers, content and uptake mechanism by the target cells, which is summarized in Table 1.

Due to the surrounding lipid membrane, EVs prevent degradation of the transported lipid mediators (e.g., eicosanoids), proteins (cytokines, chemokines, growth factors), genetic material (mRNA, long non-coding RNAs, short non-coding RNAs/miRs, nuclear and mitochondrial DNA) and organelles (e.g., mitochondria) by enzymes. EVs in the bronchoalveolar fluid (BALF) are mainly lung-specific exosomes, suggesting that exosomes predominantly serve for local signaling [52] with most cross talk taking place between alveolar epithelial cells and alveolar macrophages. Lung cells communicate intensely via EVs, which are released by respiratory cells at the apical and at the basal site, by alveolar macrophages, by other immune cells in the lungs and by fibroblasts [53]. In the healthy lung, EVs released from the macrophages down-regulate secretion of cytokines by alveolar epithelial cells. In respiratory lung diseases, EVs with higher content of pro-inflammatory cytokines, metalloproteinases and lower content of suppressor molecules are found in BALF. On the other hand are lung epithelial cell derived EVs also found in blood suggesting an exchange of EVs across the air-blood barrier and effects over longer distances. The exchange of EVs between blood and lung can explain why intravenously administered EVs can influence lung physiology. Information on the mechanism of intercellular signaling by miRs and proteins contained in EVs are available in several reviews (e.g., [52,54,55]).

EVs act on target cells either by release of soluble mediators, by receptor binding or by endocytosis [56]. While receptor-mediated uptake suggests specificity, the inter-species activity of EVs supports the hypothesis of stochastic uptake. It is possible that disease-induced alterations of the cell surface affect EV uptake. Similarly, the expression of the main uptake routes, clathrin-mediated uptake, caveolin-mediated uptake, clathrin and caveolin independent uptake, macropinocytosis and phagocytosis, may be linked to cellular differences in the uptake of EVs. Clearance of EVs from air space and blood occurs usually within minutes but with prominent inter-individual differences. In addition to various mRNAs and proteins, miR-145, miR-221, miR-133b, miR-223, miR-146a and miR-let-7c are important components of EVs for the treatment of lung diseases [57]. Despite the prominent role, which is presently attributed to miRs, calculations based on the amount of miRs in MSC-derived EVs and the possible number of EVs that could be taken up by target cells obtained concentrations, that were too low to cause relevant biological effects [58]. These data suggest that proteins are the drivers of the biological effects of EVs.

### 3.4. Differences in the Actions of MSCs and EVs

EVs due to their small size can permeate epithelial barriers. Furthermore, clearance of EVs from the organism is slower. MSCs are cleared within 24 h from the circulation but EVs were detected one hour after injection in parenchymal cells and macrophages of the damaged tissue and remained there for up to 7 days [59]. While MSCs are damaged by shear stress and retained in the pulmonary vasculature, EVs are not affects by these processes. Another advantage is the ability of storage of EVs in the absence of DMSO, which may have biological effects. Further, the absence of HLA I - and HLA II expression provides them with lower immunogenicity than MSCs, which express HLA I constitutively and HLA II after stimulation with IFN-γ [60]. EVs do not activate the complement system or induce opsonization of antigen-presenting cells like MSCs. The immune effects are due to MSCs disrupted by shear stress in the blood circulation [37]. Although also MSC-derived EVs have strong prothrombotic effects, MSCs induce more thrombosis because they also obstruct small blood vessels due to the large size of the cells and cell agglomeration [61]. Thromboembolism occurs mainly in the lung, where MSCs are retained by binding to vascular cell adhesion protein 1 (VCAM-1) of endothelial cells. Despite better penetration of epithelial barriers, EVs and conditioned media may act less beneficial because they may not able to reproduce all beneficial effects of MSCs. For instance were MSCs able to prevent ischemia-perfusion kidney damage, while conditioned media was not effective [35]. It has also been reported that exosomes were less efficient in maintaining endothelial barrier function in vitro. They acted, however, similarly efficient in a hemorrhagic shock and laparotomy induced lung injury model in vivo [62]. The lack of cell-to-cell contact and mitochondrial transfer through tunneling nanotubes or microvesicles may explain the lower efficacy [63]. As compensation for the lower efficacy, EVs can be loaded with small molecules, miRs, proteins and other macromolecules. This loading could be either occur exogenously after EV isolation or endogenously during biogenesis of the EVs [64]. Exogenous loading occurs via electroporation, simple incubation, surfactant treatment, sonication, extrusion and freeze thawing. These procedures result often in aggregation of EVs or cargo and alter physicochemical properties and morphology of the EVs. In the endogenous procedure, the parent cells are transfected or co-incubated and cultured to produce EVs. Cells can also be engineered to express the molecule of interest, which is then contained in the released EVs. More details on the different loading techniques for EVs can be obtained for instance in reviews dedicated to this topic [65,66,67]. Currently, the spectrum of loaded exosomes is limited to cytostatic agents like celastrol, paclitaxel, gemcitabine and taxol for use in cancer and EVs for few other indications such as spinal cord injury, Alzheimer disease and periodontal defects [40].

One limitation may be cell-specificity of the action because exosomes from lung spheroids outperformed EVs from MSCs. Lung-resident MSCs were shown to represent a unique population with a different phenotypic and gene expression pattern than MSCs derived from other tissues [68]. Lung MSCs compared to bone marrow MSCs expressed epithelial genes to greater extent and more remarkably differentiated to epithelial cells in retinoic acid treatment [69]. Further, some concerns remain about the potential tumor growth promoting effects of EVs. Tumor-promoting effects of MSC-derived exosomes were observed by activation of extracellular signal-regulated kinase 1/2 (ERK1/2) signaling, transfer of tumor suppressor miR-15a, miR-410, protection against cell stress, decrease of tumor cell apoptosis, exchange of MMP-2 and ecto-5′ nucleosidase and promotion of angiogenesis [70]. In addition, the immunomodulatory action of EVs may favor tumor growth.

### 3.5. Therapeutic Use of MSCs and MSC-Derived Products in Pulmonary Diseases

#### 3.5.1. Clinical Trials with MSCs

Due to the wealth of promising preclinical studies, MSCs entered clinical trials for treatment of various diseases several years ago. Table 2 shows that until 2020 the number of clinical trials focusing on pulmonary diseases was low (10–31 studies). However, this situation changed in 2020 due to the urgent need for efficient treatments of severely ill corona virus disease-19 (COVID-19) patients. COVID-19 caused by severe acute respiratory syndrome coronavirus 2 (SARS-CoV-2) started in December 2019 in China but developed to a pandemic in spring 2020. Although the virus affects numerous organs, ARDS, cardiac injury and disseminated intravascular coagulation are the main causes of death [71]. Studies published in 2020 (Table 2) identified 68–83 clinical trials for treatment of COVID-19, ARDS and IPF as main indications. Although the classification into pulmonary diseases differed between the studies, it was obvious that the pandemic more than doubled the number of registered trials on pulmonary diseases. Lists of published trials, mainly focused on ARDS induced by SARS-CoV-2, are available in recent reviews (e.g., [5,39,46,72,73]).

Although often not listed as separate disease in the analyses, BPD may be an important indication for the use of MSCs. Intratracheal administration of human umbilical cord-derived MSCs decreased severity of BDP in pre-term born infants [82] with IL-6, IL-8, metalloprotease 9 (MMP-9), TNF-α and TGF-β1 levels being reduced in the BALF. The available data suggest anti-inflammatory effects as main action of MSCs in ARDS and BPD. Unfortunately, no long-term effects are available. It is therefore unclear if the treatment can also affect the tissue repair (fibrotic response) of the disease.

A recent survey analyzed 120 studies available worldwide using stem cells, progenitor cells and exosomes in respiratory diseases in May 2020 [5]. Main indication was ARDS with 32 studies, with the majority (20 trials) being COVID-19 patients. Other important indications were BPD (21 trials), COPD/emphysema (18 trials) and IPF (9 trials). The majority were administrations of MSCs (82 trials), other stem cells accounted for 29 trials, epithelial progenitor cells for 6 trials and exosome application for 3 trials. Intravenous administration was performed in 73 trials and intratracheal administration in 18 trials. Other administration routes were chosen in the remaining trials.

An overview on nine clinical trials using MSCs for treatment of severely ill COVID-19 patients showed that MSC preparations were very heterogenous and difficult to compare [83]. While all MSCs were administered by intravenous injection, other parameters like tissue origin, manufacturer, number of donors and viability of the cells (50–95%) differed between the studies. The characterization of the cells was also not indicated in some studies. The meta-analysis reported that mortality was reduced not significantly from 43% in the controls to 25% in the treated group. Pulmonary parameters (lung injury score, tidal volume, lung compliance, ratio of arterial oxygen partial pressure (PaO_2_) to fractional inspired oxygen (FiO_2_), dependence on mechanical ventilation, stay at Intensive Care Unit) and lung condition according to imaging were improved at 5 days with no differences at longer time points. Levels of pro-inflammatory cytokines (IL-1, IL-6, TNF-α, C-reactive protein) were also reduced and IL-10 levels increased within 5 days. A press release published after this meta-analysis reported 83% survival in the Remestemcel-L-treated collective compared to 12% in the control group [84], although it has to be mentioned that this was not a randomized control trial (RCT). Importantly, beneficial effects of MSCs were seen in severe ARDS, while, similar to preclinical data, adverse effects in mild disease were reported in patients [39]. The efficacy of several stem cell products in COVID-19 pneumonia is being evaluated in ongoing trials [46]. Products include NestaCell (MSCs), CAStem (cells differentiated from clinical-grade human embryonic stem cells), Multistem (Bone marrow-derived adherent progenitor cells), RYONCIL (Remestemcel-L; culture-expanded MSCs derived from the bone marrow of an unrelated donor), XCell-UMC-Beta (Wharton-Jelly MSCs), ACT-20-MSC (MSCs from human umbilical cord tissue + conditioned media) and PLX-PAD (placenta-derived mesenchymal stromal-like cells). The majority are small trails and only in three trials ≥ 100 patients will be included.

#### 3.5.2. Studies on the Efficacy of MSC-Derived Products

Much fewer clinical trials than with MSCs have been started assessing the effects of conditioned media for the treatment of lung diseases but effects of conditioned media obtained from MSCs have been evaluated in preclinical models. The systemic review containing a meta-analysis by Emukah et al. is based on 10 studies that fulfilled the selection criteria, three on asthma, three on ARDS, two on BDP and two on PAH [85]. The following conclusions were made (i) conditioned media improved inflammation, (ii) it was equally efficacious to MSCs and (iii) the intravenous route was superior in reducing inflammation compared to the intratracheal route. The superiority of the intravenous route contrasts with other studies and will be discussed in Section 4.

Preclinical models showed promising effects of EVs isolated mainly from bone marrow-derived MSCs. The EVs demonstrated species and inter-species activity in models of ARDS, asthma, PAH and BPD [44]. Consistently seen were reductions of lung edema and of neutrophils, protein and inflammatory markers in BALF in the ARDS models, decrease of airway hyper-responsiveness and Th2/Th17 related cytokines in BALF in the asthma model and reduction of right ventricular arterial pressure, vascular remodeling and right ventricular hypertrophy in the PAH models. Similar to MSCs, EVs isolated from MSCs acted more efficient after specific pre-treatments. Pre-treatment of MSCs with ischemia or toll-like receptor 3 agonist increased the efficacy of the isolated EVs [86,87]. Compared to bacterial models, there are only few (6) in vivo studies on the effects of EVs in viral infections. Prevention of epithelial damage by secretion of KGF, Ang-1, HGF and release of EVs and stimulation of cytotoxic CD8 cells by yet unknown mediators are important in the improvement of ARDS induced by viral infection.

Few clinical trials using MSC-derived EVs for the treatment of lung diseases are also available. A variety of extracts from MSCs are available but the Food and Drug Association (FDA) ushered warnings against the use of some products (e.g., Lieveyon, Chara Biologics and RichSource Stem Cells Inc. [88]) and presently, there are no FDA-approved exosomes products. However, accelerated approval for treatment of COVID-19 pneumonia may be expected. Beneficial action with absence of adverse effects was reported after intravenous injection of bone marrow-derived MSCs (ExoFlo™) in COVID-19 induced ARDS [89]. Among the registered clinical trials, two studies will use MSC-derived exosomes (NCT04384445, NCT04376987) and one will use exosomes from COVID-19-specific T-cells from donors (NCT04389385). The donor T-cells will be challenged with viral peptide fragments in the presence of cytokines in vitro and applied by pressurized metered dose inhaler (pMDI) in the early phase of COVID-19 pneumonia. Zofin™ (Organcell™ Flow) is derived from amniotic fluid and contains approximately 400 billion EVs per milliliter. Five COVID-19 patients had been treated with Zofin under the FDA’s emergency Investigational New Drug (eIND) Program in a phase I/II trial and, according to the producer company, significant improvements in the condition of these patients were achieved [90]. The ExoFlo™ exosome preparation was granted expanded access by the FDA in the treatment of COVID-19 [91]. It contains 10 billion of 30–150 nm particles, various growth factors and is provided as sterile filtered solution [92]. Similar to MSCs the main administration of EVs is intravenous injection. However, one phase I/II clinical trial will assess the efficacy of nebulized allogenic MSC-exosomes in ARDS (NCT04602104). Further, phase I clinical trials have also been initiated to assess the effects of nebulized exosomes in critically ill COVID-19 patients.

## 4. Intravenous versus Inhalation Route of Delivery of MSCs and MSC-Derived Products

It may be surprising that administration of MSCs and MSC-derived products by inhalation is seen as an alternative to intravenous injection because formulation as aerosol is more complicated than preparation for intravenous injection. There are, however, specific problems linked to intravenous injection of MSCs, which might be prevented by inhalation.

Intravenously administered MSCs ended to 67% up in the lungs and elimination of MSCs from the lungs was reported as 24 h after intravenous delivery [32,93]. By contrast, after intraperitoneal injection in mice, human umbilical MSCs were detected on day 3 and day 8 in various organs (lungs, spleen, kidney, liver, colon, heart, lymph nodes, testes, seminal vesicles and urinary bladder) [94]. The trapping effect in the lungs is due to the fact that stem cells and progenitor cells are too big to pass the pulmonary circulation [95]. Labelled MSCs administered intravenously to mice were viable for 24 h in the lungs but no viable cells reached other organs, such as liver [96]. Further, the infused MSCs did not leave the capillary bed. In principle, MSCs should be able to extravasate because they can bind to E-selectin of endothelial cells, which is involved in coordinated rolling and extravasation. However, the expression, compared to leukocytes is very low and the migration capacity of cultured MSCs compared to other cells, poor [37]. Extravasation may be increased upon upregulation of receptors for chemokines CCRs2-4 of MSCs in inflamed tissues but still the large (15–30 µm) cell size, the large nucleus and the intrinsic nuclear lamina properties hinder the ability of MSCs to migrate [97]. The high content of lamins A/C was suggested as the main reason for the lack of extravasation and the fast clearance of intravenously administered MSCs in vivo. Consistent with this hypothesis, MSCs with down-regulated lamins A/C remained longer in the lung than the not manipulated cells. The low passage of endothelia is specific for the cultured MSCs; endogenous MSCs are smaller and can efficiently traffic via systemic circulation [98]. This problem could be solved by inhalation as administration route because Kim et al. showed that MSCs delivered to specific regions of rat, porcine and human lungs by intratracheal administration, attached to the respiratory epithelium [99]. Due to methodological limitations, however, their fate over prolonged time could not be assessed. Another negative aspect of intravenous administration of MSCs is their thrombotic effect through formation of cell aggregates and complement activation [25,62]. Intratracheal delivery may be further useful for patients needing extracorporeal membrane oxygenation (ECMO) because MSCs attach to the membrane oxygenator fibers [60]. This could be prevented by infusing the cells prior to ECMO in patients not needing continuous high-flow ECMO. If, however, continuous high-flow ECMO is needed, only intratracheal delivery would be an option.

One meta-analysis, focused on preclinical models, reported that intratracheal instillation of MSCs was better for treatment of ARDS than intravenous and intraperitoneal administration [100]. Intratracheal injected MSCs also performed better than MSCs applied by intravenous injection in murine COPD models [101]. Emukah et al. [85], on the other hand, reported smaller effects upon inhalation in one study each for asthma, ARDS and BPD in preclinical models. Since the studies using intravenous and inhaled MSCs were not performed by the same group of researchers, the comparison is subjected to bias caused by use of different MSC preparations and sources and differences in the disease models.

Endotracheal application of cells has been performed mainly in the two human pathologies BPD and IPF. Intratracheal application of Pneumostem^®^ to premature infants was reported to be safe and to reduce the severity of the disease [82]. Similarly, endotracheal application of MSCs in IPF patients was safe and deterioration of lung function was stopped during the 12 months of observation [102]. In a follow-up trial, progression of the disease in these patients was observed after 24 months. Since no control group was included in the study, it is not clear if application of the cells was efficient [103].

Most of the potential advantages of inhalation (lower shear stress, better permeation of epithelial barriers and less prothrombotic action) play a minor role for EVs. Preclinical data reported similar potency of MVs delivered by intravenous and intrachaeal route in ARDS [104]. This is not surprising because EVs are expected to cross epithelial barriers easily [87]. EVs injected intravenously into mice first reached spleen and liver and subsequently distributed to gastrointestinal tract and lungs [105]. Permeation for exosomes across epithelial barriers (e.g., blood-brain barrier, gastrointestinal barrier) has been shown for endogenous and exogenous (Bacillus subtilis-derived) exosomes [106]. The transport is assumed to be better when parental cells and barrier forming cells are similar. For instance crossed Caco-2-derived EVs Caco-2 monolayers five times better than milk-derived EVs [107]. The greater stability of the EVs may enable the development of formulations that can be self-administered by the patient by inhaler or nebulizer, which would enable long-term treatment. This option would be useful to prevent progression of tissue remodeling seen in the repair phase of ARDS, in IFP and in PAH.

**Figure 4:**
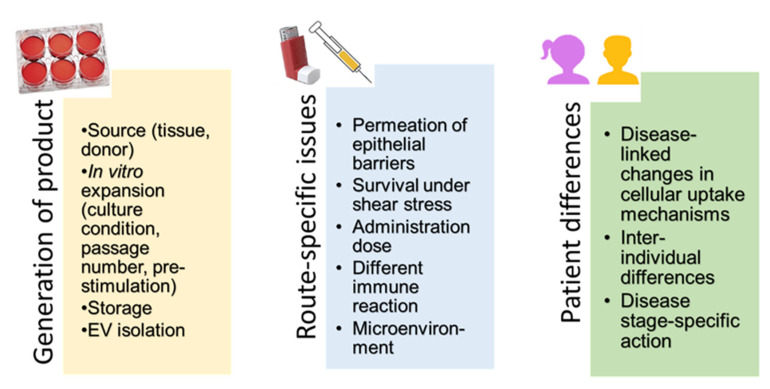
Overview of important sources for variability between study results.

Presently, it is difficult to identify an advantage of administration by inhalation because there are many differences between the studies. They comprise variations in generation of the products, uncertainties in administration-related issues and differences between patients and disease stages. These factors are summarized in Figure 4. It may be hoped that the awareness of these problems and available guidelines for standardization increase comparability of results from ongoing clinical trials and enable better assessment of the effects of MSCs and MSC-derived products in pulmonary diseases.

## 5. Administration of MSCs and MSC-Derived Products

### 5.1. Production of MSCs and MSC-Derived EVs as Medicinal Products

A great part of differences between study results has been attributed to differences in the products. Elimination of the main sources for product variability is of key importance for better inter-study comparison. Medicinal products have to be produced in sufficient amounts with reproducible purity, identity, quantity, potency and sterility [108]. This is difficult for MSCs, which differ between donors, tissues (e.g., adipose tissue, bone marrow, dental pulp, etc.), passage number (cell senescence), seeding density and culture conditions (bioreactors, stimulation with LPS, growth factors, protein- and miR modulation, small molecule inhibitors or hypoxia) and storage (e.g., freezing in DMSO) [57]. Another problem is heterogeneity of the cultured MSCs. It was found that smaller MSCs grow more rapidly and age less than the larger cells [109]. Further, this population of MSCs was retained in the lung to a smaller extent and acted more efficient in a murine elastase-induced emphysema model. Based on this data, it would be useful to employ size separation of cells and use only the small ones for therapeutic interventions. However, cell separation (e.g., sedimentation, density gradient, microfluidic separation, etc.) may introduce additional variation in the final product [110]. Yield of EVs is influenced by seeding density and passage number of the parent cells [58], where both high passage number and cell density have an adverse effect on particle production. Further, EVs from senescent cells act also less effective on the target cells. EV production can be increased by use of bioreactors that increase EV production by a factor of 40 compared to conventional culture, probably due to higher shear stress and compressive forces. Hypoxia (1–2% O_2_), regular filtration or ultracentrifugation have similar stimulating effects on EV release. Sequential centrifugation, which assumedly causes decreased re-uptake of the EVs, used for the production of clinical grade EVs. Further, the yield of MSC-derived EVs can be increased by addition of cytochalasin B [111]. Particles produced this way are more homogenous, act angiogenic and are taken up by cells to a higher amount than EVs produced without stimulation. It is not clear if the immunomodulatory potential differs between stimulated and not stimulated MSCs. A summary of the sources for variation of MSCs and MSC-derived products and the state of standardization in provided in Table 3.

Based on the guidelines of the International Society for Extracellular Vesicles on minimal information for studies of extracellular vesicles (MISEV) the following information from the parent cells should be listed: tissue source, number of dead cells, incubation time until harvest, passage number, seeding density, extent of confluence at harvest, culture volume, culture vessel/bioreactor, surface coating, oxygen or other gas tension and stimulations/pre-treatments [112]. Specific additions to the cell culture medium or their absence during the harvesting, have to be listed, for example, fetal bovine serum (FBS), platelet lysate, pituitary extracts, bile salts and so forth. Use of EV-depleted medium (method or provider, including lot number) has to be indicated. The protocol for isolation has also to be described because the cytokine content of exosomes isolated from conditioned media by four different methods was significantly different [113]. Important information on the EVs includes three positive protein markers for EVs, including at least one transmembrane lipid-bound protein, one negative protein marker and absence of contaminating proteins (e.g., serum-derived material). Description of surface morphology by high resolution techniques (e.g., scanning electron microscopy (SEM), atomic force microscopy (AFM) or high-resolution microscopy), biophysical properties (by e.g., size-nanoparticle tracking analysis (NTA), flow cytometry); fluorescence properties (by e.g., fluorescence correlation spectroscopy, flow cytometry) and chemical composition (by e.g., Raman spectroscopy) is essential. In addition, particle number, total protein, lipid and RNA content have to be reported. The guidelines of the European Medical Agency (EMA) for biopharmaceuticals are similar but list the requested information more precisely [114]. Physicomechanical and immunochemical properties, biological activity, purity and impurities need to be indicated in the characterization. Amount, viability and phenotype of the parent cells has to be provided as well. Information on the EVs should include size by NTA, total protein and ratio of protein/particles or CD9/CD63/CD81-positive particles to protein. Expression of CD9, CD63, CD83 and tumor susceptibility gene 101 protein (TSG101) as EV markers, CD44, CD73, CD90 and CD105 as MSC markers and the absence of the immune cell markers CD14, CD34 and CD45 characterize clinical grade EVs [58]. For batch release of exosomes, the expression of CD9, CD63, CD81, TSG101, ALG-2 (apoptosis-linked gene 2)-interacting protein X (Alix) and ganglioside GM1, negative endotoxin, negative mycoplasma, absence of viral enrichment and sterility are requested. EVs can be stored without addition of DMSO but may aggregate. To prevent aggregation and clumping the addition of stabilizers like glucose, sucrose or trehalose, is suggested [40].

Usually 5–10% of the protein is contained in vesicles, which means that the preparation is rather a particle-enriched secretome fraction than a pure vesicle product. The exosome-specific issues are related to sterility since currently used enrichment procedures also enrich viruses and high-throughput filter technology favors contamination with endotoxin. More information of the regulatory requirement for the production of MSC-derived EVs is available elsewhere (e.g., [114]). Biological activity needs in vitro or in vivo testing. For characterization of the biological activity, it would be essential to know the active ingredients of the EVs (e.g., proteins, miRs, other molecules).

### 5.2. Methods for Topical Administration to the Lungs

Local delivery of the lungs can be achieved by invasive and non-invasive techniques. Invasive techniques apply suspensions of cells (or other active ingredients) to specific regions of the lungs and little preparation or formulation of the cells is needed. In preclinical studies, the most common way is using the MicroSprayer^®^ Aerosolizer. Aerosolization by MicroSprayer^®^ Aerosolizer did not reduce viability of the MSCs [115]. The aerosolized MSCs attenuated airway inflammation and structural airway changes in ovalbumin sensitized rabbits [116].

In the clinical setting, cells would be administered to humans using flexible bronchoscopy, which is a minimally invasive procedure and similar to surfactant delivery to newborns. Both applications involve deposition of therapeutic materials on the airway surfaces via liquid plugs traveling through the pulmonary airways [99]. The investigator can perform endoscopic examination of the tracheobronchial tree and deliver the product precisely to the desired region. Since anesthesia is necessary, the intervention is not indicated for routine treatment [117]. Regarding uniformity of the distribution pattern, intratracheal administration is inferior to inhalation by spontaneously breathing patients [1].

Non-invasive administration is driven by the inspiratory airflow of patients and requires formulation of the product to allow release from the device and deposition in the airways. Available types of inhalers are pressurized metered dose inhalers, soft mist inhalers, dry powder inhalers and ultrasonic, jet and vibrating mesh nebulizers. Cells are sensitive to the physical and chemical stress related to formulation and aerosolization and, therefore, metered dose inhalers and dry powder inhalers are not feasible for administration [118]. However, cells can be aerosolized using soft mist inhalers, jet nebulizers and vibrating mesh nebulizers. According to data by Averyanov et al., compressor nebulization performed better than ultrasonic nebulization and mesh nebulization [119]. In the bleomycin-induced lung fibrosis model, survival of MSCs after aerosolization with either ultrasound, jet or mesh nebulizers and after intravenous injection were compared [120]. Viability of MSCs was highest after nebulization with jet nebulizer and effects in vivo were better after treatment with the nebulized cells than with the injected cells. The better performance of the nebulized cells could be explained by an activation of the MSCs similar to pre-conditioning of MSCs by other stimuli. It has been shown that priming by addition of Toll ligand receptors LPS or poly I:C, cytokines (TNF-α, IL-1β, interferon-γ), culture in hypoxic condition or in 3D improved survival and immune response of the administered MSCs [121]. Overall, too few data are currently available to recommend the most suitable aerosolization technique for MSCs.

From the formulation aspect, EVs are more suitable for inhalation treatment than MSCs due to the better stability against aerosolization by nebulizer, greater stability upon freezing and the possibility to use excipients/stabilizers and cryoprotectants (trehalose, lactose, mannitol) [45]. Secretome and exosomes tolerate the aerosolization techniques of all inhalers. Conditioned media from bone marrow and umbilical cord MSCs nebulized by Aerogen^®^ Solo nebulizer maintained its antimicrobial properties with LL-37, hepcidin and lipocalin-2 as the active peptides [122]. Further, secretome and exosomes from lung spheroid cells, administered to mice and rats by jet nebulizer, improved lung histology and lung function in silica-induced fibrosis [123]. The use of EVs instead of MSCs has the additional advantage that uniform delivery of cells by inhalation is less complicated for exosomes than for cells [124]. The fact that nebulized MSC-derived exosomes caused beneficial effects in minor cases of COVID-19 patients demonstrated the suitability of aerosolized exosomes for the treatment of pulmonary diseases [125].

Recruiting/active clinical trials use exosomes delivered either by nebulizer (NCT04276987, NCT04473170, NCT04491240, ChiCTR2000030261, NCT04602104) or pressurized metered dose inhaler (NCT04389385) with indication of ARDS. MSCs isolated from adipose tissue, autologous non-hematopoietic peripheral blood stem cells, not specified MSCs and specific T cells are indicated as exosome sources in these studies. More information on these trials is available in recent reviews and on ClinicalTrials.gov (accessed on 30 December 2020) (e.g., [1,72,126]). The small number of studies presently available does not allow identification of the number of inhaled MSCs to achieve optimum effects and of potential differences in the immune response to allogenic MSCs between intravenous and inhalation administration.

## 6. Conclusions

MSCs and MSC-derived EVs have shown promising effects in the treatment of respiratory diseases. While MSCs can be conveniently administered by intravenous injection, their survival in the organism is short and their ability to cross the air-blood barrier questionable. Thrombus formation in the pulmonary vasculature and retention in filter for extracorporeal membrane oxygenation are additional disadvantages of intravenous administration of MSCs. Topical administration to the lung is feasible when using minimally invasive procedures like bronchoscopy. The non-invasive administration by inhalers poses the problem that an aerosolization technique preserving sufficient cell viability has to be chosen.

MSC-derived EVs represent a promising option for both intravenous and inhalation treatment. As they cross better epithelial barriers, are more stable and display less prothrombotic effects, the advantage of administration by inhalation appears smaller. If, however, formulations for use with conventional inhalers or nebulizers existed, long-term treatment by a non-invasive route would be possible. 

## Figures and Tables

**Table 1:** Characterization of MSC-derived vesicles according to origin, size, surface marker, content and uptake mechanism by the target cells (according to [51]). Abbreviations: ESCRT, endosomal sorting complex required for transport; TSG101, tumor susceptibility gene 101 protein.

Type of Extracellular vesicle	Type of Secretion of Vesicle	Size of Vesicle	Surface Marker of the Extracellular Vesicle	Content of the Extracellular Vesicles	Uptake by Target Cell
Apoptotic bodies	Membrane blebbing	500–2000 nm	Phosphatidylserine, calreticulin, calnexin	Proteins, lipids, nuclear fragments, organelles	Phagocytosis
Microvesicles	Blebbing	100–1000 nm	Integrins, selectins, CD40	Proteins, lipids, ncRNAs, mRNA	Fusion
Exosomes	Exocytosis from multivesicular bodies	40–100 nm	Tetraspanins (CD9, CD63, CD81), ESCRT, TSG101, flotillin, annexin	Membrane and cytoplasmic proteins, lipids, ncRNAs, mRNA, MHC molecules, receptors	Endocytosis

**Table 2:** Overview of clinical trials listing the fraction (%) of studies evaluating pulmonary diseases among all trials or the fraction of specific lung diseases, when only trials on pulmonary diseases were included.

Year of Publication	Number of Trials Analysed	% of (Specific) Pulmonary Diseases in the Analysed Trials	Reference
2015	339 (all indications)	3 (pulmonary diseases)	[74]
2015	516 (all indications)	6 (pulmonary diseases)	[6]
2016	493 (all indications)	5 (pulmonary diseases)	[75]
2017	109 (all indications)	10 (pulmonary diseases)	[76]
2019	49 (pulmonary diseases)	BPD (37), IPF (14), ARDS (29), asthma (4), COPD (4)	[77]
2020	767 (all indications)	7 (pulmonary diseases)	[78]
2020	62 (COVID-19)	N.a.	[79]
2020	73 (ARDS, COVID-19)	ARDS (57), COVID-19 (22), ARDS/COVID-19 (21)	[1]
2020	16 (COVID-19)	N.a.	[80]
2020	83 (pulmonary diseases)	COVID-19 (47), pulmonary fibrosis (10), ARDS (10), asthma (2), COPD (7)	[81]
2020	68 (pulmonary diseases)	COVID-19 (45), ARDS (15), COPD (15), asthma (3), IPF (9)	[39]

**Table 3:** List of MSCs and MSC-derived products with content, sources for variations and comments regarding standardization.

Product	Content	Source of Variation	Comments
Stem cells (e.g., mesenchymal stem cells)	Viable cells	Donor, tissue source, composition of culture media, cell density, passage number, pre-culture conditions	Tissue source and pre-conditioning can be reported
Conditioned media	Soluble proteins, extracellular vesicles	As for stem cells plus culture time until collection, volume and composition of the collection medium	Isolation method can be standardized. There are no guidelines for characterization of the conditioned media
EVs/exosomes	Cytokines, growth factors, signaling lipids, mRNAs, regulatory miRs	As for conditioned media plus isolation method and storage condition of EVs	Recommendations for characterization of exosomes are available

## Data Availability

Not applicable.
